# Dihydromyricetin Alleviates Non-Alcoholic Fatty Liver Disease by Modulating Gut Microbiota and Inflammatory Signaling Pathways

**DOI:** 10.4014/jmb.2406.06048

**Published:** 2024-11-20

**Authors:** Limin Kang, Xiaolong Ma, Fakun Yu, Lei Xu, Li Lang

**Affiliations:** 1 Department of Hepatobiliary and Pancreatic Surgery, Puer People’s Hospital, Puer 665000, P.R. China; 2 Department of Reproductive Genetics Center, Puer People’s Hospital, Puer 665000, P.R. China; 3 Department of Outpatient, Puer People’s Hospital, Puer 665000, P.R. China

**Keywords:** Dihydromyricetin, non-alcoholic fatty liver disease, intestinal flora, toll-like receptor 4/nuclear factor κB, inflammatory response, liver protection

## Abstract

Non-alcoholic fatty liver disease (NAFLD) is a prevalent chronic liver condition that is strongly linked to gut microbiota imbalance and chronic inflammation. This study aims to explore the preventive effects of dihydromyricetin (DHM) on NAFLD by modulating the intestinal flora and the TLR4/NF-κB signaling pathway. Fifty male C57BL/6J mice were randomly assigned to five groups: a normal control group, a model group, and three DHM treatment groups receiving low (500 mg/kg), medium (750 mg/kg), and high doses (1,000 mg/kg). NAFLD was induced using a high-fat diet, and DHM was administered for 8 weeks. ELISA measured serum levels of LPS, IL-1β, and TNF-α, while Western Blot assessed liver expression of TLR4 and NF-κB p65. Changes in intestinal flora composition were analyzed using high-throughput 16S rRNA sequencing. The results showed that DHM treatment significantly reduced serum levels of LPS, IL-1β, and TNF-α, decreasing the liver expression of TLR4 and NF-κB p65. Intestinal flora analysis indicated a notable increase in beneficial bacteria, especially in the medium and high-dose groups. DHM treatment also significantly improved liver pathology, reducing fat deposition and inflammatory cell infiltration. In conclusion, DHM effectively prevents the progression of NAFLD by improving gut microbiota balance and suppressing inflammatory signaling pathways.

## Introduction

Non-alcoholic fatty liver disease (NAFLD) is among the most common chronic liver diseases globally, characterized by widespread hepatocellular steatosis unrelated to alcohol or other explicit liver injury factors [1]. As a complex chronic multisystem disorder, the occurrence and progression of NAFLD are influenced by various factors, including genetic predisposition, environmental elements, individual metabolic status, and intestinal microbiota balance [2]. Often coexisting with other metabolic disorders such as type 2 diabetes mellitus, hypertension, and hypercholesterolemia, NAFLD manifests systemic metabolic disturbances with common symptoms like abnormal glucose tolerance and dyslipidemia [3]. Furthermore, liver function in NAFLD patients may deteriorate gradually, leading to non-alcoholic steatohepatitis (NASH), liver fibrosis, and potentially cirrhosis and hepatocellular carcinoma [4].

The prevalence of NAFLD is steadily rising due to the global increase in lifestyle-related diseases such as obesity and diabetes [5]. Due to its widespread impact, the prevention and management of NAFLD are crucial in clinical practice and integral to global public health policies [6, 7]. Although current treatment methods primarily focus on lifestyle adjustments such as increasing physical activity, modifying the dietary structure, and weight reduction, these measures are often challenging to sustain long-term and have limited efficacy [8-10]. Therefore, in-depth research on NAFLD can aid in understanding its complex pathogenic mechanisms and facilitate the development of more effective preventive and intervention strategies, ultimately enhancing patients' quality of life and reducing the incidence of related complications [11].

Recent studies have revealed a strong association between intestinal flora and chronic metabolic diseases such as NAFLD and diabetes [12]. The intestinal microbial structure and composition in NAFLD patients and mice show significant alterations compared to healthy individuals [13, 14]. By modulating the intestinal flora in NAFLD mouse models, researchers have observed improvements in maintaining intestinal barrier function and regulating key molecules such as short-chain fatty acids and lipopolysaccharides (LPS), ultimately alleviating NAFLD symptoms [15]. Furthermore, disruption of the intestinal mucosal barrier may lead to the entry of bacterial endotoxins like LPS into the bloodstream [16], subsequently activating TLR4 and its downstream NF-κB signaling pathway, resulting in the release of inflammatory factors, including interleukin-1β (IL-1β) and tumor necrosis factor-α (TNF-α) [17]. This inflammatory response plays a crucial role in the onset and progression of NAFLD.

Dihydromyricetin (DHM) is a natural flavonoid compound abundant in Hovenia which has been demonstrated to possess antioxidant, anti-inflammatory, and antibacterial properties, as well as bioactivity in regulating glucose and lipid metabolism [18, 19]. Previous studies suggest that DHM may have a preventive or therapeutic effect on chronic metabolic diseases like NAFLD [20, 21]. However, the exact mechanisms by which DHM prevents and treats NAFLD remain unclear, including whether it modulates the intestinal flora and subsequently regulates the TLR4/NF-κB signaling pathway to impact NAFLD [22-24].

This study aims to investigate the preventive and therapeutic effects of DHM on NAFLD by modulating the intestinal flora and inhibiting the TLR4/NF-κB signaling pathway. We will assess the impact of DHM using a mouse NAFLD model induced by a high-fat diet, aiming to deepen the understanding of its mechanisms at the molecular level and explore the biological effects of different DHM doses. The findings of this study may identify novel targets for intervening in the prevention and treatment of NAFLD, providing a scientific basis for clinical practice to offer patients safer and more effective disease management strategies, thereby improving their quality of life and reducing the risk of related complications.

## Materials and Methods

### Drugs and Reagents

In this study, DHM was the primary intervention drug provided by Shanghai Biotechnology Co., Ltd.,(China) with a purity of 98%. The high-fat diet (H10045, 45% fat energy ratio) and control diet (H10010, 10% fat energy ratio) for mice were purchased from Beijing Huafukang Biotechnology Co., Ltd., (China) All mice underwent a minimum one-week acclimatization period on a standard diet before the experiment. The reagents used in the experiment included assay kits for total cholesterol (TC), triglyceride (TG), high-density lipoprotein cholesterol (HDL-c), low-density lipoprotein cholesterol (LDL-c), and liver function-related assays for alanine aminotransferase (ALT), aspartate aminotransferase (AST), alkaline phosphatase (ALP), and γ-glutamyl transferase (γ-GT), all procured from Nanjing Jianjian Biotechnology Co., Ltd., (China). Additionally, ELISA assay kits for measuring serum levels of LPS, IL-1β, and TNF-α were sourced from Shanghai Kaibo Biotechnology Co., Ltd. TLR4 and NF-κB p65 expressions in the liver were analyzed using the Western Blot method, with the necessary rabbit anti-mouse TLR4 and NF-κB p65 antibodies supplied by Wuhan DeboShi Biological Engineering Co., Ltd. (China) [25].

### Animal Grouping and Modeling

Fifty 3-4-week-old male C57BL/6J mice were obtained from the Experimental Animal Center of Kunming University of Science and Technology for this study. All animal handling procedures were approved by the Institutional Ethical Committee of the Experimental Animal Center of Kunming University of Science and Technology (Approval No: kmlg2021-0042). Following the method described in the literature [26], a high-fat diet-induced NAFLD in the mice. After one week of acclimatization, 10 mice were randomly assigned to the normal control group (NG) and fed a standard diet, while the remaining 40 mice were fed a high-fat diet to induce NAFLD. After 4 weeks, the NAFLD mice were randomly divided into four groups, each containing 10 mice. These groups comprised the low-dose DHM Group (DLG), medium-dose DHM Group (DMG), High-Dose DHM Group (DHG), and Model Group (MG), where the MG continued to consume the high-fat diet without DHM supplementation. The doses of DHM in the groups were 500 mg/kg/day, 750 mg/kg/day, and 1,000 mg/kg/day, respectively. The intervention period lasted for a total of 8 weeks. At the end of the study, after a 12-h fast, the mice were weighed and anesthetized intraperitoneally with 100 mg/kg pentobarbital sodium, and blood samples were collected via retro-orbital puncture. Subsequently, the mice were euthanized, and their liver and epididymal fat tissues were collected and weighed. The organ index was calculated by dividing the weight (grams) by the final body weight (grams) and multiplying by 100%. The liver samples were divided into two parts: the left lobe was preserved in 4% paraformaldehyde solution for histopathology and other examinations, while the remaining liver tissue was stored at -80°C for Western blot and related tests. The cecum contents were collected into cryotubes and stored at -80°C for analysis of intestinal microbiota.

### Biochemical Marker Analysis

A series of biochemical markers were assessed to comprehensively evaluate the effects of DHM on an NAFLD mouse model. It included levels of TC, TG, HDL-c, and LDL-c in serum, analyzed using an automated biochemical analyzer (Myriad BS-420, Shenzhen Myriad Biomedical Electronics Co., Ltd., China) following the manufacturer's guidelines [27]. Additionally, liver function was evaluated by measuring the activities of ALT, AST, ALP, and γ-GT in serum. Regarding inflammatory markers, LPS, IL-1β, and TNF-α in serum were determined via the ELISA technique.

### Western Blot Analysis of TLR4 and NF-κB p65 Protein Expression in the Liver of Mice

To thoroughly analyze the effects of DHM on the expression of TLR4 and NF-κB p65 proteins in a mouse model of NAFLD, the Western Blot technique was utilized in this study. The experimental procedures were as follows: Initially, liver tissue samples weighing 100 mg each were ground into powder in liquid nitrogen, followed by lysis buffer (Cat#GK10023, Good Laboratory Practice bioscience, USA) to obtain the total protein solution. This solution was centrifuged at 4°C to collect the supernatant as the total protein sample. The protein concentration was determined using the BCA protein quantification kit (Cat#PC0020, Solarbio). Subsequently, SDS-PAGE gel electrophoresis was performed, and the proteins were transferred from the gel to a PVDF membrane (Cat#ISEQ00010, Solarbio) using wet transfer at a constant current of 300mA for 30 minutes or 200mA for 1 hour. The membrane was then blocked with 5% non-fat milk powder (Cat#LP0033B, Solarbio) and incubated overnight at 4°C with TLR4 antibody (Abcam, Cat#ab22048) and NF-κB p65 antibody (Cat#8242S, Cell Signaling Technology, USA) at a 1:500 dilution each, with GAPDH antibody (Cat#97166S, Cell Signaling Technology) as the internal control at a dilution of 1:1000. The following day, after washing the membrane with TBST at room temperature, it was incubated at room temperature on a rocking platform for 1 h with secondary antibodies conjugated with horseradish peroxidase against mouse IgG (Cat#A0286, Beyotime, China) at a dilution of 1:3000 or rabbit IgG (Cat#A0277, Beyotime, China) at a dilution of 1:2000. After another round of washing, the ECL detection system was used for chemiluminescent visualization, and the bands were observed with the Quantity One imaging system. Subsequent relative quantification analysis was conducted using Image Plus 6.0 image analysis software [28, 29]

### Pathological and Histological Analysis

A comprehensive pathological and histological analysis was conducted to assess the impact of DHM on the liver pathology of mice with NAFLD [30]. The fixed tissues underwent dehydration, transparency, and embedding in paraffin, with a slice thickness of 5 micrometers. Liver sections were stained with hematoxylin and eosin (H&E) to observe changes in tissue structure and pathological conditions. Oil Red O staining reagent (Cat#C0157S, Beyotime) was utilized to further evaluate fat deposition in the liver, explicitly labeling lipid substances and presenting lipid droplets within liver cells in red. All stained sections were observed and captured under an optical microscope. Furthermore, a comparison of tissue sections between the treatment and control groups was performed to assess the potential protective effects of DHM on hepatic pathological changes.

### Analysis of Gut Microbiota

The gut microbiota analysis was conducted by extracting DNA from mice's cecum contents [31]. A microbial DNA extraction kit (Qiagen, Cat. No. / ID: 51704, German) was used to ensure the quality and purity of DNA suitable for subsequent high-throughput sequencing. Specific primers targeting the V3 and V4 variable regions of the 16S rRNA gene were used for PCR amplification. The commonly used primer pair 341F/805R, widely applied in many microbiome studies [32], was utilized. The sequences are as follows: 341F: 5'-CCTACGGGNGGCWGCAG-3'; 805R: 5'-GACTACHVGGGTATCTAATCC-3'. The amplified products were then purified and used for library construction. Subsequently, high-throughput sequencing was performed on the Illumina NovaSeq platform to achieve sufficient sequencing depth to cover gut microbiota diversity. The sequencing data were processed using QIIME 2 (version 2021.2) for quality control and chimera removal, and the DADA2 plugin (version 1.14) was employed for denoising the sequences. OTU clustering was based on 97% sequence similarity and assigned to the corresponding genera. We used the SILVA database (version 138) as the reference database for taxonomic classification, further validating classification results using the Greengenes database. Additionally, α-diversity and β-diversity of the microbial community were analyzed to assess the richness and evenness of the gut microbiota of mice and the differences in microbial composition among different experimental groups. These analyses were used to quantitatively evaluate the impact of DHM on gut microbial balance, particularly in terms of changes in the abundance and diversity of beneficial bacterial groups.

### Statistical Analysis

The data were analyzed using spss 25.0 software. Before analysis, normality and homogeneity of variance tests were conducted for the measured data. For normally distributed data, one-way analysis of variance (ANOVA) was used to compare differences between different groups, followed by post hoc LSD tests to analyze specific intergroup differences. Non-normally distributed data were analyzed using the Kruskal-Wallis non-parametric test. Experimental results were presented as mean ± standard deviation (SD). Graphs were generated using GraphPad Prism 9.0 software, with significance levels of *p* < 0.05 and *p* < 0.01 considered statistically significant [33].

## Results

### Significant Reduction of Mouse Body Weight and Fat Deposition by DMH Intervention

Fifty male mice, aged 3-4 weeks, were divided into two groups. Ten mice were randomly assigned to the NG and fed a standard diet, while the remaining 40 mice were fed a high-fat diet to induce NAFLD. After 4 weeks, the NAFLD mice were randomly divided into four groups: the DLG, the DMG, the DHG, and the MG, with 10 mice in each group. The model group continued to receive the high-fat diet without DHM supplementation.

**Fig. 1:**
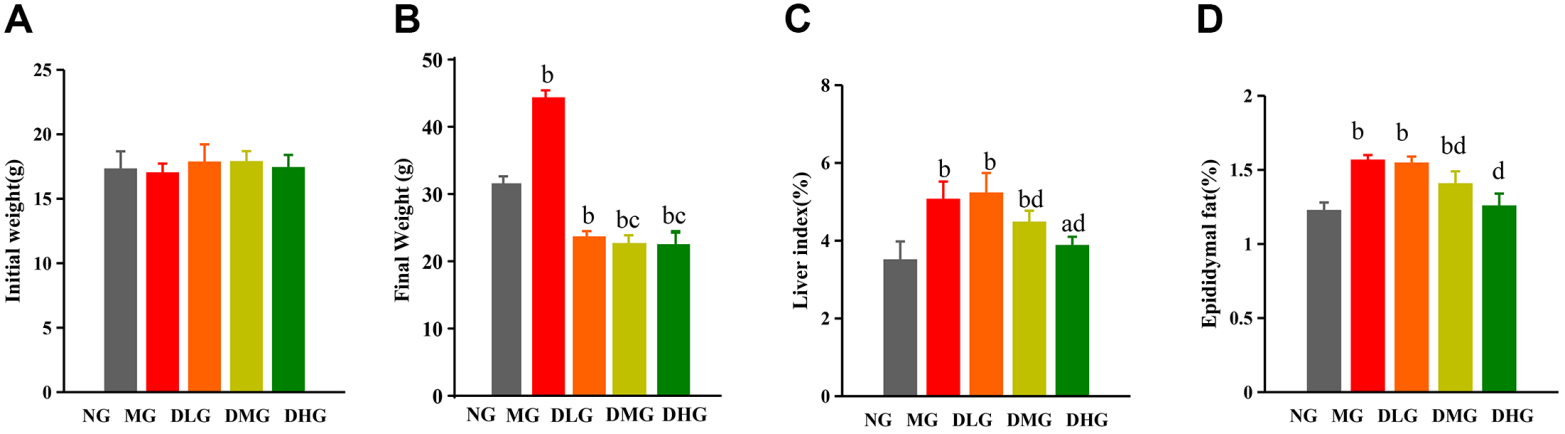
Effects of DHM on mice's body weight, organ, and adipose indexes. (**A**) Initial body weight levels across different groups (**B**) Final body weight levels across different groups (**C**) Liver index levels across different groups (**D**) Epididymal fat index levels across different groups. Data are presented as mean ± SD (*n* = 10 or 8). Statistically significant differences compared to the NG are denoted as (^a^*p* < 0.05, ^b^*p* < 0.01). Statistically significant differences compared to the MG are denoted as (^c^*p* < 0.05, ^d^*p* < 0.01).

At the end of the experiment, mice in the NG exhibited healthy signs, such as smooth fur, normal activity, and regular food and water intake, with no recorded deaths. In contrast, mice in the MG showed significant health issues, including dull fur, reduced activity, increased food intake, and two deaths during the experiment. Mice in the DMH-treated groups, including the DLG, DMG, and DHG, did not experience any fatalities. Compared to the NG, mice in the MG significantly increased body weight, liver weight, and epididymal fat index (Fig. 1, *p* < 0.01), indicating successful induction of an NAFLD model by a high-fat diet. However, mice in the DHM-treated groups showed significantly reduced body weight and fat deposition compared to the MG mice, with the effects being more pronounced in the DMG and DHG groups (Fig. 1, *p* < 0.01 or *p* < 0.05). Specifically, the DMG and DHG mice showed significantly lower body weight, liver weight, and epididymal fat index than the MG. These results demonstrate that DMH effectively mitigates weight gain and fat deposition induced by a high-fat diet in mice, particularly at higher doses, highlighting the significant potential of DMH in improving body weight and fat deposition in a NAFLD mouse model.

### DHM Effectively Improves Lipid Abnormalities

**Fig. 2:**
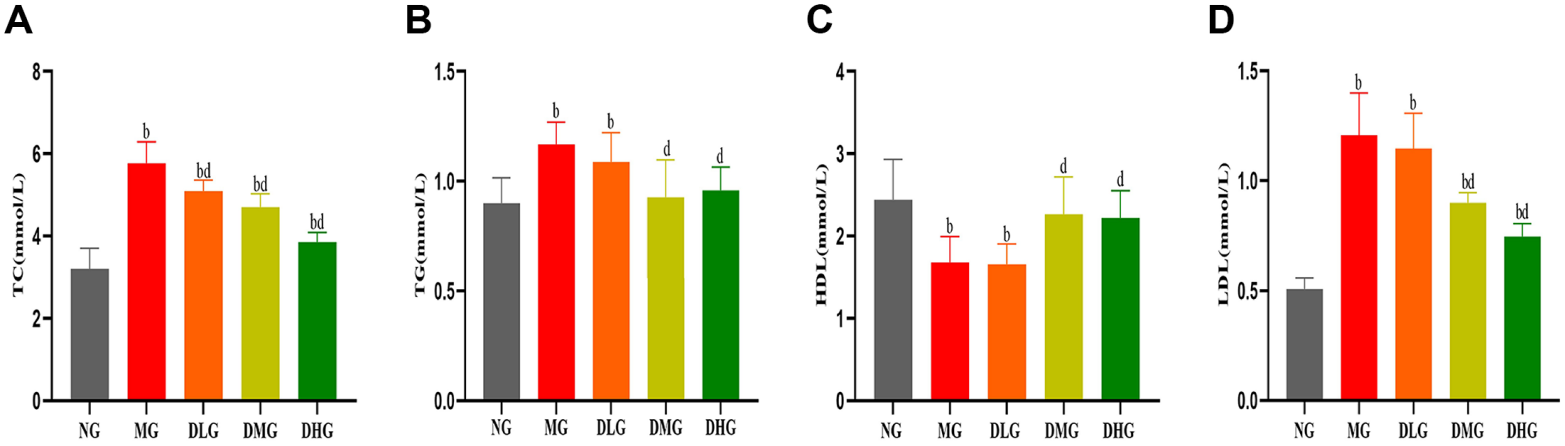
Influence of DHM on serum lipids in mice with NAFLD. (**A**) TC levels in different groups (**B**) TG levels in different groups (**C**) High-density lipoprotein levels in different groups (**D**) Low-density lipoprotein levels in different groups. Data are presented as mean ± SD (*n* = 10 or 8). Statistically significant differences from the NG are indicated as (^a^*p* < 0.05, ^b^*p* < 0.01). Statistically significant differences compared to the MG are indicated as (^c^*p* < 0.05, ^d^*p* < 0.01).

The analysis of lipid levels in mice revealed that the MG exhibited significant increases in serum TC, TG, and LDL-c levels, while HDL-c levels significantly decreased (Fig. 2A-2D, *p* < 0.01), indicating successful induction of lipid metabolism disorder by a high-fat diet. In contrast, the DHM-treated group demonstrated a significant improvement in lipid levels, with DHM treatment significantly reducing the levels of TC, TG, and LDL-c in mice while significantly increasing HDL-c levels, particularly in the DMG and DHG (Fig. 2A-2D, *p* < 0.01). Compared to the MG, the medium and high dose groups showed significant reductions in TC and TG levels and increased HDL-c levels (Fig. 2A-2D, *p* < 0.01). These results indicate that DHM effectively reverses lipid metabolism disorders induced by a high-fat diet, restoring lipid levels to near-normal states.

### DMH Significantly Improves Liver Function Markers

**Fig. 3:**
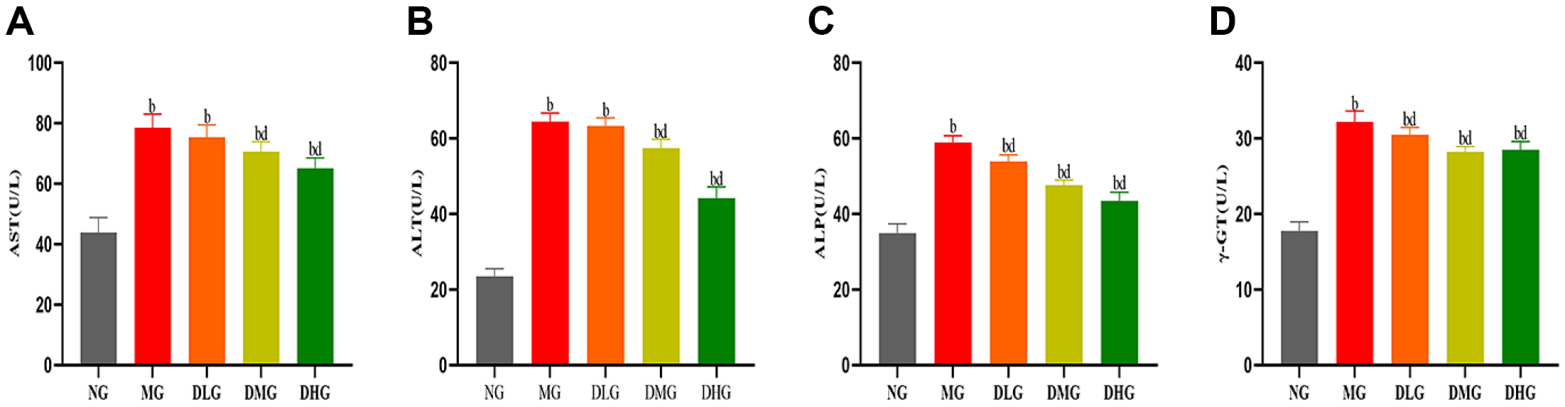
Impact of DHM on serum hepatic function in mice with NAFLD. (**A**) Serum AST levels in different groups (**B**) Serum ALT levels in different groups (**C**) Serum ALP levels in different groups (**D**) Serum γ-GT levels in different groups. Data are expressed as mean ± SD (*n* = 10 or 8). Statistically significant differences compared to the NG are denoted as (^a^*p* < 0.05, ^b^*p* < 0.01). Statistically significant differences compared to the MG are denoted as (^c^*p* < 0.05, ^d^*p* < 0.01).

At the end of the experiment, mice in the MG exhibited a significant deterioration in liver function, characterized by significant increases in AST, ALT, ALP, and γ-GT levels (Fig. 3A-3D, *p* < 0.01), indicating severe liver damage due to a high-fat diet. However, DHM treatment significantly improved these liver function markers. Particularly in the DMG and DHG, mice showed significant reductions in serum AST, ALT, ALP, and γ-GT levels, presenting a marked improvement compared to the MG (Fig. 3A-3D, *p* < 0.01 or *p* < 0.05). Although the DLG also improved, the effect was not as significant as observed in the DMG and DHG. These results suggest that DHM effectively alleviates liver damage caused by a high-fat diet and significantly enhances liver function. Overall, DHM demonstrates significant protective effects in improving liver function in NAFLD mice.

### Suppressive Effect of DHM on Systemic and Liver-Specific Inflammation in Mice

**Fig. 4:**
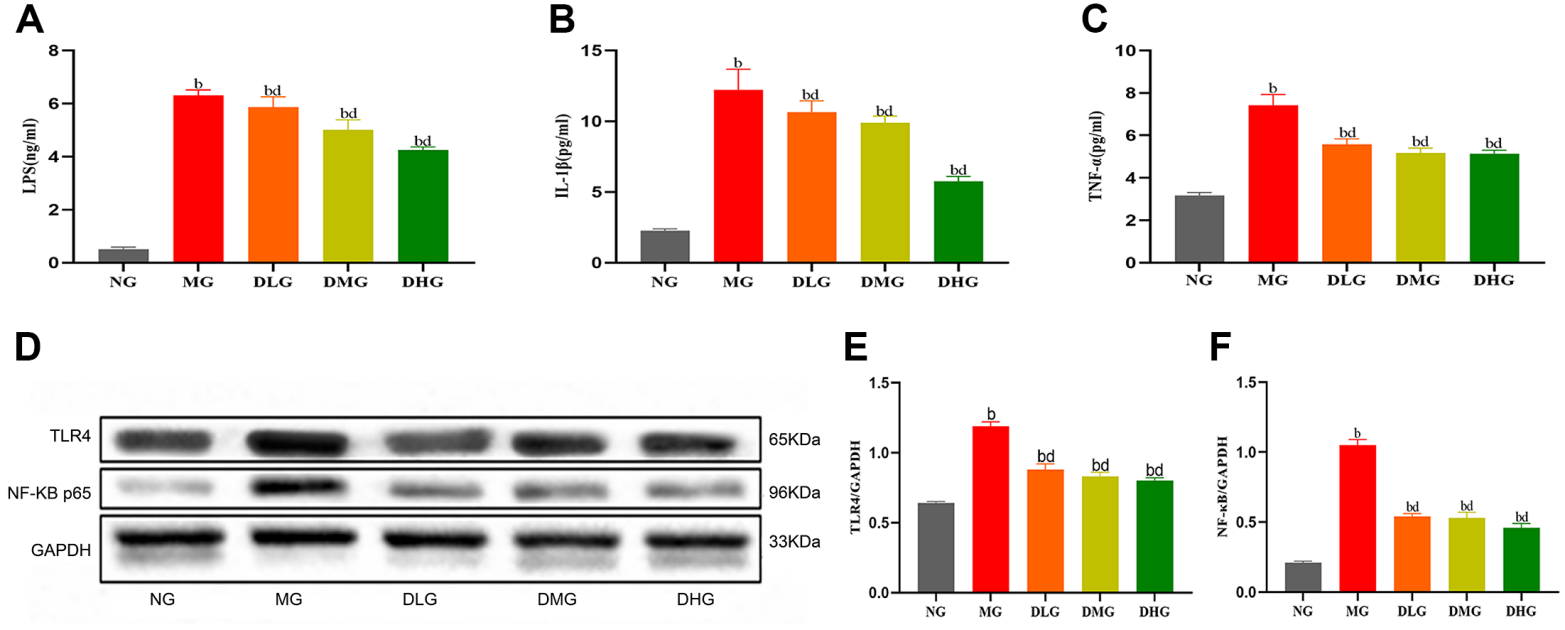
Influence of DHM on serum inflammatory factors and hepatic TLR4 and NF-κB levels in mice with NAFLD. (**A**) Serum LPS levels in different group (**B**) Serum IL-1β levels in different groups (**C**) Serum TNF-α levels in different groups (**D**) Immunoblot of TLR4 and NF-κB proteins in mouse liver (**E**) Hepatic TLR4 protein levels in different groups (**D**) Hepatic NF-κB protein levels in different groups. Data are presented as mean ± SD (*n* = 10 or 8). Statistically significant differences from the NG are identified as (^a^*p* < 0.05, ^b^*p* < 0.01). Statistically significant differences compared to the MG are identified as (^c^*p* < 0.05, ^d^*p* < 0.01).

In comparison to the NG, mice in the MG exhibited significantly elevated levels of serum inflammatory factors, specifically showing a notable increase in concentrations of LPS, IL-1β, and TNF-α (Fig. 4A-4C, *p* < 0.01), indicating a pronounced systemic inflammatory response induced by the high-fat diet. In contrast, mice in the DHM treatment groups, notably the DMG and DHG, showed significant reductions in these inflammatory factor levels (Fig. 4A-4C, *p* < 0.01). Although the DLG also demonstrated some inhibitory effects, the effect was not as significant as observed in the medium and high-dose groups.

Further exploration was conducted to understand DHM's impact on inflammatory cytokines by examining the relationship between the TLR4/NF-κB signaling pathway and the expression of LPS, IL-1β, and TNF-α. Western Blot analysis revealed a significant increase in the expression of TLR4 and NF-κB p65 proteins in liver tissues of mice in the MG (Fig. 4D-4F, *p* < 0.01 or *p* < 0.05), indicating the activation of the TLR4/NF-κB signaling pathway by the high-fat diet, further promoting the occurrence of inflammatory responses. The DHM treatment groups, particularly in the medium and high doses, showed significant downregulation in the expression levels of TLR4 and NF-κB p65 proteins (Fig. 4D-4F, *p* < 0.01 or *p* < 0.05), with a certain degree of downregulation also observed in the low-dose group. These findings suggest that DHM mitigates the inflammatory response induced by a high-fat diet by inhibiting the activation of the TLR4/NF-κB signaling pathway. Overall, DHM demonstrates a significant role in alleviating systemic and liver-specific inflammation induced by a high-fat diet, as evidenced by the reduction in serum levels of LPS, IL-1β, and TNF-α and the inhibition of the expression of TLR4 and NF-κB p65 proteins in liver tissues. DHM effectively blocks the activation of inflammatory signaling pathways, thereby reducing the inflammatory response.

### DHM Improves Hepatic Tissue Pathological Features

**Fig. 5:**
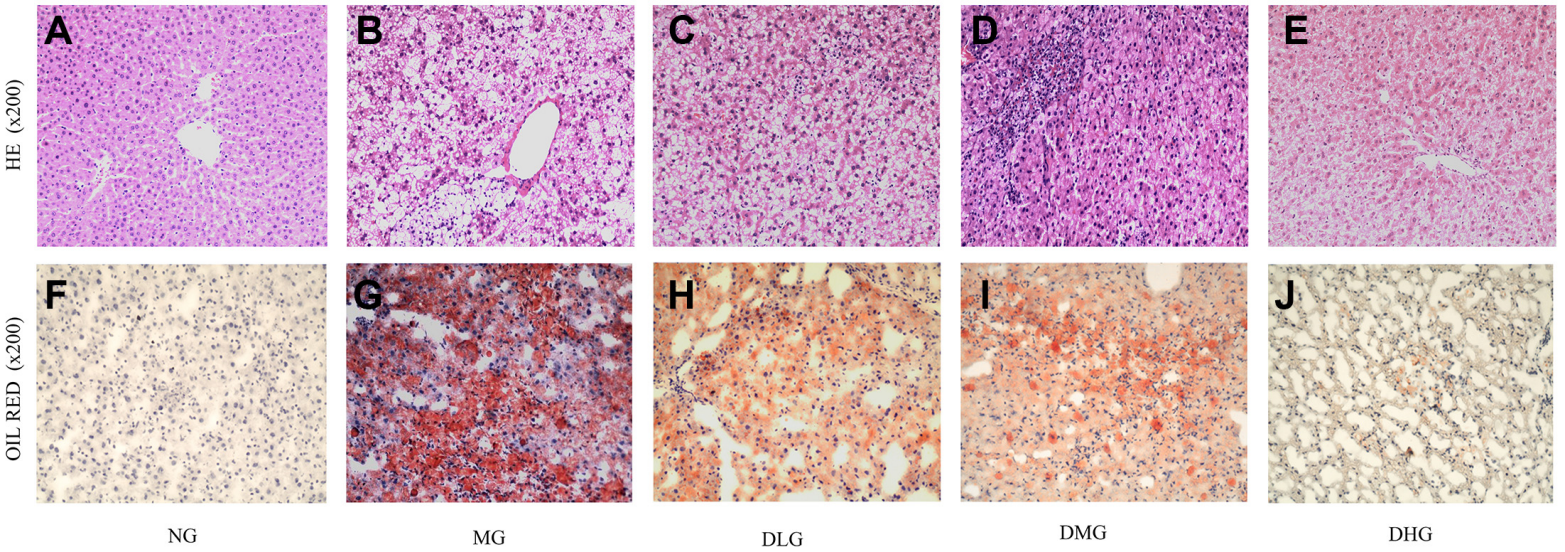
Effect of DHM on hepatic pathological changes in mice with NAFLD. (**A-E**) Hepatic sections stained with H&E in different groups (×200 magnification) (**F-J**) Hepatic sections stained with Oil Red O in different groups (×200 magnification).

Histological staining of liver tissues revealed that mice in the NG exhibited well-preserved liver tissue structure, with clear hepatic lobule outlines and hepatocytes arranged radially, indicating a healthy liver condition. In contrast, liver tissues of mice in the MG displayed substantial pathological changes, including diffuse vacuolization around the central vein, significant hepatocyte enlargement, and severe fat deposition, indicative of successful induction of NAFLD by the high-fat diet. When excessive fat is consumed, the liver struggles to effectively process it, accumulating fat within hepatocytes and resulting in steatosis. Under these conditions, hepatocytes become noticeably enlarged, and vacuolar changes can be observed around the central vein [34]. These pathological alterations further confirmed the damaging effect of the high-fat diet on liver tissues. However, following treatment with DHM, significant improvements were observed in the hepatic tissue pathology of the mice. Specifically, mice in the DHM treatment groups, especially in the DMG and DHG, exhibited liver tissue restoration closer to normal. H&E staining revealed well-arranged hepatocytes, clear hepatic lobule structures, and significantly reduced fat deposition and inflammatory cell infiltration in these treatment groups (Fig. 5A-5E). Oil Red O staining results further confirmed a significant reduction in the number of lipid droplets in the livers of mice in the DHM treatment groups (Fig. 5F-5J), indicating a protective effect of DHM against hepatic steatosis. Oil Red O is a specific dye that detects fat within tissues or cells. When a significant amount of fat is present in liver tissue, this dye binds to the fat, producing a distinct red coloration. Therefore, the liver of mice in the MG group stained a deep red, indicating significant fat accumulation and more severe steatosis [35].

Although the DLG of mice showed some improvement in liver tissue, the results were not as significant as those in the medium and high-dose groups. Specifically, the liver tissues of mice in the DLG still showed residual fat deposition and mild inflammatory cell infiltration; however, these features were notably reduced compared to the MG. These outcomes indicate that DHM effectively alleviates the pathological liver tissue damage induced by a high-fat diet through various mechanisms. DHM may exert its protective effects on the liver by modulating the intestinal flora, inhibiting the TLR4/NF-κB signaling pathway, and reducing the expression of inflammatory factors.

### DHM Modulates Intestinal Flora and Promotes Beneficial Bacteria Growth

**Fig. 6:**
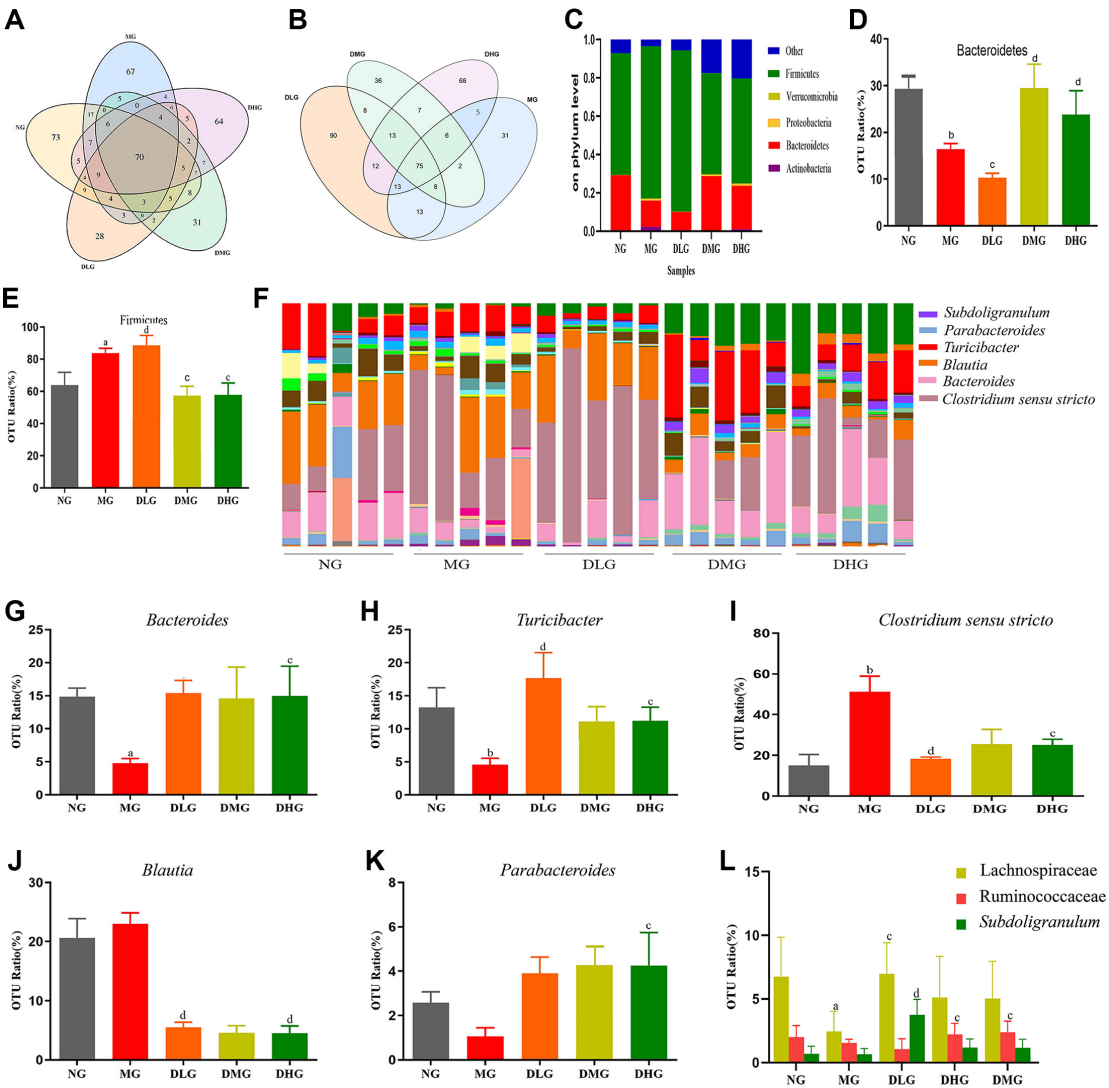
Impact of DHM on intestinal flora changes in mice with NAFLD. (**A**) Venn diagram of OTUs of gut microbiota among different experimental mouse groups. It shows the overlap of gut microbiota OTUs between the Normal Group (NG, orange), Model Group (MG, blue), Low-dose DHM Group (DLG, yellow), Medium-dose DHM Group (DMG, green), and High-dose DHM Group (DHG, purple). (**B**) Venn diagram of OTUs of gut microbiota among different experimental mouse groups. It displays the overlap of gut microbiota OTUs between the Low-dose DHM Group (DLG, yellow), Medium-dose DHM Group (DMG, green), High-dose DHM Group (DHG, purple), and Model Group (MG, blue). (**C**) Phylum-level changes in gut microbiota across different groups. (**D**) Abundance of *Bacteroidetes* expression in different groups. (**E**) Abundance of *Firmicutes* expression in different groups. (**F**) Genus-level changes in gut microbiota across different groups. (**G**) Abundance of *Bacteroides* expression in different groups. (**H**) Abundance of *Turicibacter* expression in different groups. (**I**) *Clostridium* sensu stricto expression is abundant in different groups. (**J**) Abundance of *Blautia* expression in different groups. (**K**) Abundance of *Parabacteroides* expression in different groups. (**I**) Abundance of expression of two different bacterial families (Lachnospiraceae and Ruminococcaceae) and one bacterial genus (*Subdoligranulum*) in different groups.

Through high-throughput 16S rRNA sequencing, we analyzed the composition and structure of the gut microbiota in different experimental groups of mice. Fig. 6A shows the number and overlap of OTUs among the NG, MG, and DHM treatment groups (DLG, DMG, and DHG). The number of unique OTUs in the MG group (67) is higher than that in the DHM treatment groups and the NG group, suggesting that a high-fat diet may have impacted the gut microbiota structure. After DHM treatment, the DLG group had 28 unique OTUs, the DMG group had 31, and the DHG group had 64, indicating that these specific microbial communities may be associated with the dose-specific response to DHM treatment. The 70 shared OTUs represent the core microbiota present across all groups. It suggests that DHM treatment, at varying doses, partially restored the changes in the gut microbiota induced by the high-fat diet.

In Fig. 6B, the overlap of OTUs between the MG group and the different DHM dose groups (DLG, DMG, DHG) is further compared. With increasing doses of DHM, the overlap of OTUs between the DHM-treated groups and the MG group also increased. For example, the high-dose DHM group (DHG) shared 75 OTUs with the MG group, suggesting that high-dose DHM may have a potential positive effect on restoring the gut microbiota structure. Additionally, the unique OTUs found in the DLG group (90) and the DMG group (36) indicate that DHM treatment may have exerted a dose-dependent regulatory effect on microbial diversity. These findings suggest that DHM could somewhat alleviate the potential impact of a high-fat diet on the gut microbiota. In addition, Fig. S1 presents the differences in gut microbiota α-diversity (PD Whole Tree and Observed Species Rarefaction Curves) (Fig. S1A-S1B) and β-diversity (Unweighted and Weighted UniFrac distance heatmaps)(Fig. S1C-S1D) among the groups. These analyses further support the potential role of DHM in restoring gut microbiota imbalances induced by a high-fat diet.

Fig. S1C illustrates the structural differences in the gut microbial communities across the treatment groups, with β-diversity evaluated through principal component analysis (PCA). The PCA results indicate a clear separation between MG and NG along the PC1 (35.32%) and PC2 (19.78%) dimensions, suggesting that a high-fat diet caused significant alterations in the microbial community structure. In contrast, the DHM-treated groups, especially DMG and DHG groups, show reduced distances between their sample coordinates and those of the NG group along the PC1 and PC2 dimensions, reflecting that DHM treatment partially restored the balance of the gut microbial community structure. Furthermore, Fig. S1D presents a heatmap of weighted UniFrac distances, which quantitatively assesses the β-diversity differences among the groups. The weighted UniFrac distance between the MG and NG groups is relatively large, indicating that the high-fat diet-induced significant phylogenetic differences. In contrast, the weighted UniFrac distances between the DHM-treated groups (especially DMG and DHG) and the NG group are smaller, suggesting that these treatment groups share greater phylogenetic and community structural similarity with the NG group. These findings support the significant role of DHM in alleviating gut microbial dysbiosis caused by a high-fat diet.

At the phylum level, the intestinal flora of the mice in all groups primarily comprises Bacteroidetes, Proteobacteria, Firmicutes, Actinobacteria, and Cyanobacteria (Fig. 6C). Compared to the NG, the relative abundance of Firmicutes in the intestinal flora of MG mice significantly increases, while the relative abundance of Bacteroidetes decreases significantly, which is closely associated with intestinal flora imbalance and the development of metabolic syndrome (Fig. 6C-6E, *p* < 0.05 or *p* < 0.01). Following DHM treatment, the relative abundance of Bacteroidetes in the intestinal tract of mice in the DMG and DHG significantly increases compared to the MG, while the relative abundance of Firmicutes notably decreases, suggesting that DHM treatment may improve the balance of gut microbiota in mice (Fig. 6D-6E, *p* < 0.05 or *p* < 0.01).

At the genus level, in comparison to the NG, the abundance of beneficial genera such as *Bacteroides* and *Turicibacter* in the intestines of MG mice significantly decreases, while the abundance of harmful genera like *Clostridium* and *Blautia* significantly increases (Fig. 6F-6J, *p* < 0.05 or *p* < 0.01). Conversely, mice in the DHM treatment group, especially in the medium and high dose groups, exhibit a marked restoration of beneficial genera, with significant increases in the abundance of *Bacteroides* and *Parabacteroides* (Fig. 6G-6K, *p* < 0.05 or *p* < 0.01). These changes indicate that DHM treatment may improve the balance of gut microbiota in mice by enhancing the abundance of beneficial microbes and reducing the proportion of harmful ones. Notably, the relative abundance of other beneficial gut microbiota, such as the Lachnospiraceae family, Ruminococcaceae family, and the *Subdoligranulum* genus (which belongs to the Ruminococcaceae family), was significantly increased in the DHM-treated mice. These microbiota are closely associated with the host's health (Fig. 6l, *p* < 0.05 or *p* < 0.01).

## Discussion

DHM exhibits a significant reduction effect on the levels of LPS, IL-1β, and TNF-α in serum, which not only aligns with previous research findings, demonstrating its role in regulating inflammation, but also further confirms its ability to exert this effect through modulation of intestinal flora [36-38]. By reducing body weight and lowering liver and testicular fat indices, DHM can potentially combat metabolic disruptions [22, 23]. Furthermore, it improves lipid levels and liver function indicators and reduces pathological changes in the liver, such as fat accumulation and cellular inflammation [39]. DHM demonstrated significant therapeutic effects in improving the histopathological features of liver tissue in NAFLD mice, with the effects being particularly pronounced at medium and high doses. This series of biological effects not only highlights the multifunctionality of DHM in the prevention and treatment of NAFLD but also reveals its broad potential in inhibiting the progression of this disease [40, 41]. These results further highlight the potential of DHM to improve metabolic health in NAFLD mice by modulating the gut microbiota. In summary, DHM demonstrated significant efficacy in improving lipid metabolism in NAFLD mice, supporting its potential as a non-pharmacological intervention for regulating blood lipid levels.

In regulating inflammatory signaling pathways, DHM effectively suppresses hepatic inflammation by significantly inhibiting the expression of TLR4 and NF-κB p65 [42, 43]. This mechanism aligns with previous research suggesting that DHM can modulate the transmission of inflammatory signals by balancing the intestinal flora [44, 45]. Specifically, DHM reduces the binding of the TLR4 receptor to its ligand LPS, thereby inhibiting the activation of the downstream NF-κB signaling pathway, leading to reduced production of pro-inflammatory cytokines like IL-1β and TNF-α [46]. Additionally, DHM enhances intestinal barrier function through various pathways, reducing intestinal permeability and lowering the likelihood of endotoxins entering the bloodstream [47, 48]. This mechanism controls local hepatic inflammation and exerts a systemic anti-inflammatory effect by reducing systemic inflammatory responses [39, 49]. The systemic anti-inflammatory effect of DHM provides theoretical solid support for its clinical use in treating NAFLD and other inflammation-related metabolic diseases [50]. For instance, DHM can reduce the inflammatory burden in chronic diseases such as diabetes, cardiovascular diseases, and others [51-53]. Through further research validation, DHM has the potential to serve as an effective therapeutic option in managing these diseases that significantly impact human health.

This study validates the significant effects of DHM in regulating intestinal flora and inhibiting inflammatory signaling pathways and expands the current literature. Previous research has predominantly focused on the antioxidant and anti-inflammatory properties of DHM, particularly its effects in in vitro experiments and acute liver injury models [54]. However, there is relatively limited research on the mechanisms of action of DHM in chronic liver diseases such as NAFLD, specifically its regulatory role in intestinal flora [40, 55]. Using high-throughput 16S rRNA sequencing technology, the researchers provide a detailed analysis of the impact of DHM on the intestinal flora of NAFLD mice. The findings reveal a notable reduction in the abundance of inflammation-associated Firmicutes and an increase in beneficial Bacteroidetes and Lactobacillaceae, aligning with existing research but offering more specific insights. Furthermore, this study systematically explores for the first time how DHM regulates the TLR4/NF-κB inflammatory signaling pathway through intestinal flora modulation, thereby alleviating hepatic inflammation and improving liver function. These findings not only deepen our understanding of the mechanisms of DHM in NAFLD but also offer new theoretical foundations and research directions for its clinical application. In contrast with previous studies, this research significantly supplements the knowledge about the mechanisms of DHM in chronic liver diseases, laying the groundwork for developing NAFLD treatment strategies based on intestinal flora regulation.

This study identifies a significant regulatory effect of DHM on the intestinal flora, notably reducing the abundance of inflammation-associated Firmicutes while increasing the proportion of Bacteroidetes. This discovery starkly contrasts with the typical pattern of intestinal flora imbalance in NAFLD patients, highlighting the potential of DHM in restoring a healthy microbial community structure. Specifically, the use of DHM significantly enhances the abundance of Lactobacillaceae (*Bacteroides*) and Bacteroidetes (*Parabacteroides*), beneficial probiotic groups that aid in the breakdown of complex carbohydrates to produce short-chain fatty acids like butyrate, essential for intestinal health. Through these changes, DHM improves nutrient breakdown and energy production in the gut and effectively reduces intestinal inflammation, maintaining gut homeostasis. These results indicate that DHM can modulate intestinal microbiota through multi-layered mechanisms, thereby exerting therapeutic effects on NAFLD.

Despite achieving significant findings, this study has several limitations. Firstly, it was conducted solely using a mouse model. While these results can, to some extent, reflect the therapeutic effects of DHM on NAFLD, differences in pathophysiological mechanisms between animal models and human diseases may affect the generalizability of the results and require further verification. Secondly, the study lacked a positive control group, making it challenging to comprehensively assess the relative efficacy of DHM compared to other established treatment methods. Additionally, metabolic profiling analysis was not included, limiting our understanding of the changes in gut microbial metabolites under the influence of DHM; further metabolomics studies could reveal the specific mechanisms of action of DHM. Lastly, the study had a relatively short duration, which hindered the assessment of the long-term efficacy and safety of DHM. Future research should involve more extended observation periods to ensure the safety and effectiveness of long-term DHM use.

Future research on NAFLD should be deepened in various aspects. Firstly, there is an urgent need for human clinical trials to validate the efficacy and safety of DHM in NAFLD patients. Specifically, attention should be paid to the long-term effects and potential side effects of different doses of DHM. Secondly, large-scale metabolomics and metagenomics studies should be conducted to comprehensively elucidate the specific mechanisms of DHM in regulating intestinal flora and metabolic pathways. These studies will help identify new biomarkers, leading to more precise treatment methods. Additionally, future research should explore the combined effects of DHM with other therapeutic approaches, such as dietary adjustments, weight management, and other drug therapies, to achieve better comprehensive treatment outcomes. Lastly, researchers should also focus on the potential application of DHM in other metabolic diseases, such as type 2 diabetes mellitus and cardiovascular diseases, expanding the clinical application scope of DHM through systematic studies to bring health benefits to more patients.

## Conclusion

**Fig. 7:**
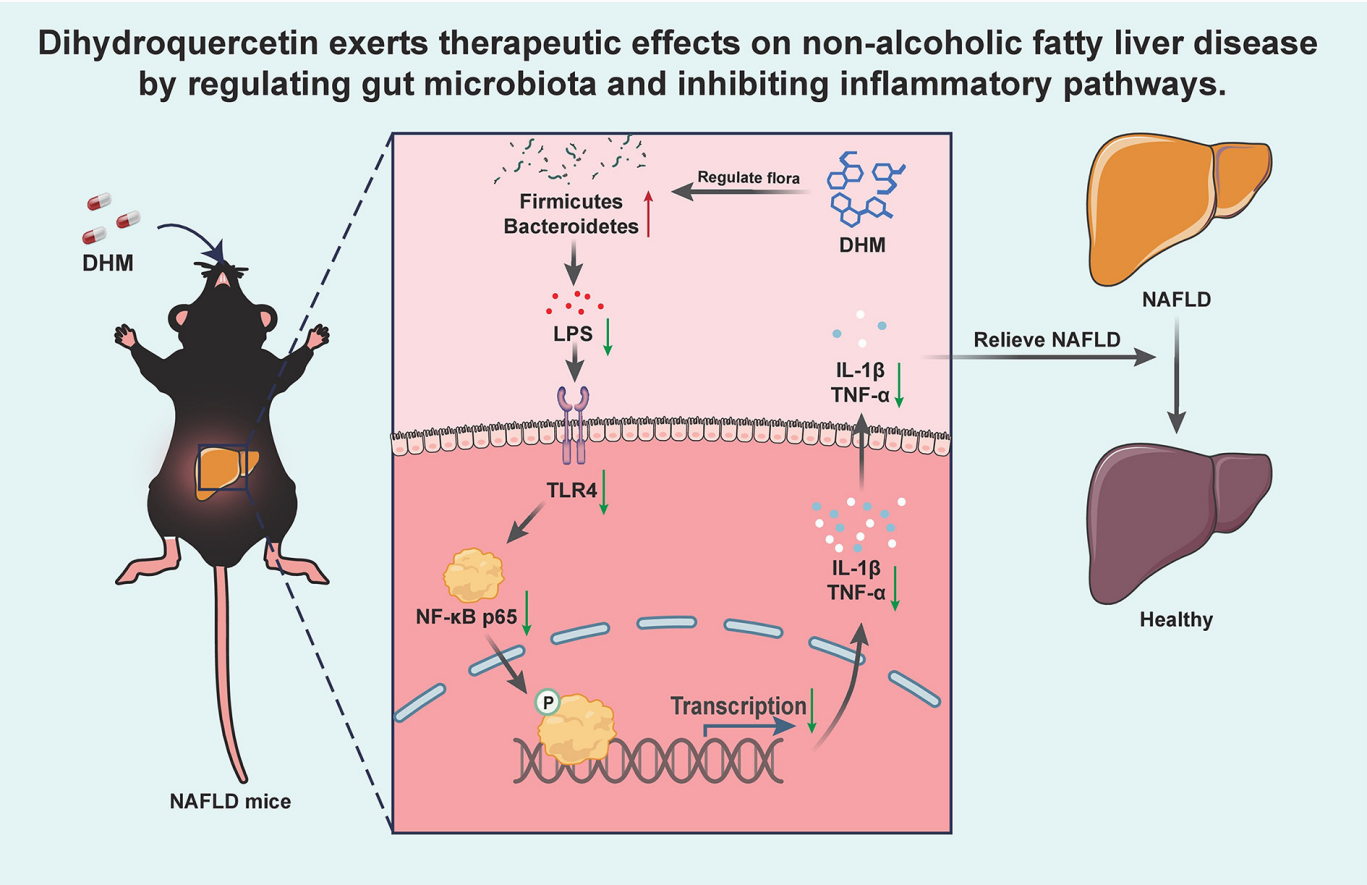
The therapeutic effect of DHM on NAFLD through modulating intestinal flora and inhibiting inflammatory pathways.

In this study, DHM significantly improved the hepatic pathological status and inflammation levels in a mouse model of NAFLD by modulating the intestinal flora and inhibiting the TLR4/NF-κB signaling pathway. It was indicated by improved blood lipid levels and liver function, reductions in the serum inflammatory factors LPS, IL-1β, and TNF-α, and downregulation of TLR4 and NF-κB p65 expression in the liver. Additionally, DHM treatment increased the abundance of beneficial gut bacteria, suggesting its potential role in improving intestinal microbial balance (Fig. 7).

## Supplemental Materials

Supplementary data for this paper are available on-line only at http://jmb.or.kr.
