# Supplementary figures and images for: Dihydromyricetin Alleviates Non-Alcoholic Fatty Liver Disease by Modulating Gut Microbiota and Inflammatory Signaling Pathways
Source: J Microbiol Biotechnol. 2024 Nov 20;34(12):2637–47. doi: 10.4014/jmb.2406.06048 (PMC11729546; doi:10.4014/jmb.2406.06048)

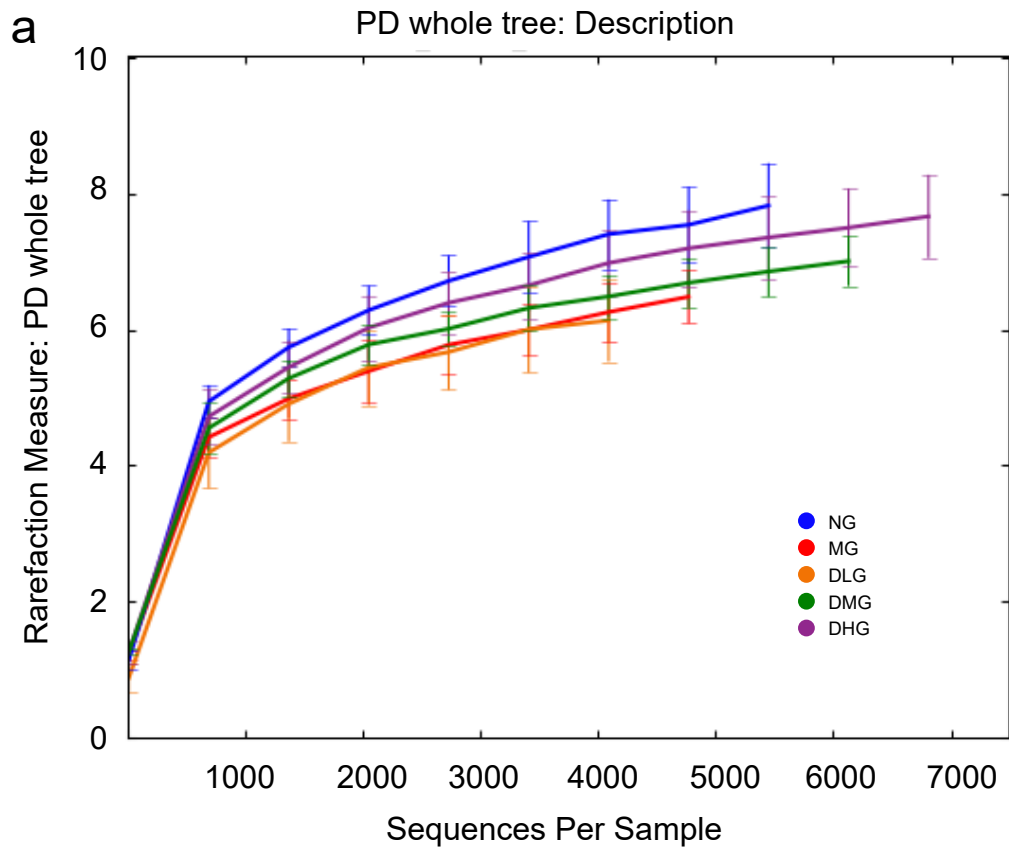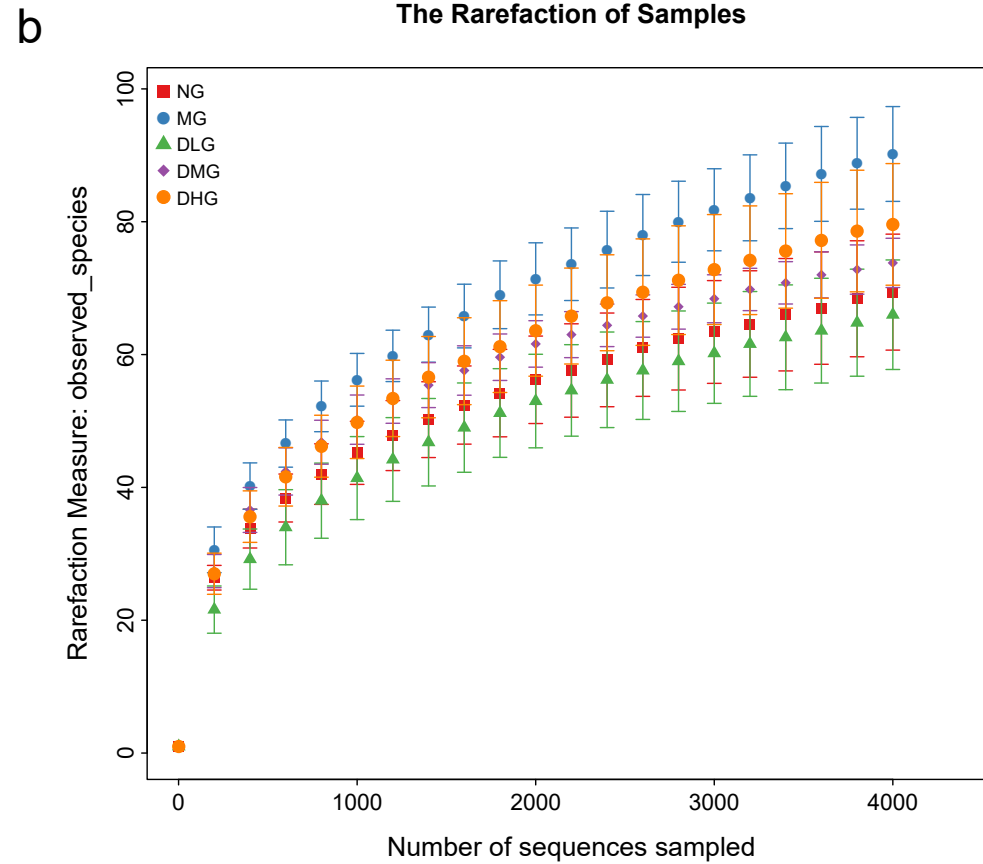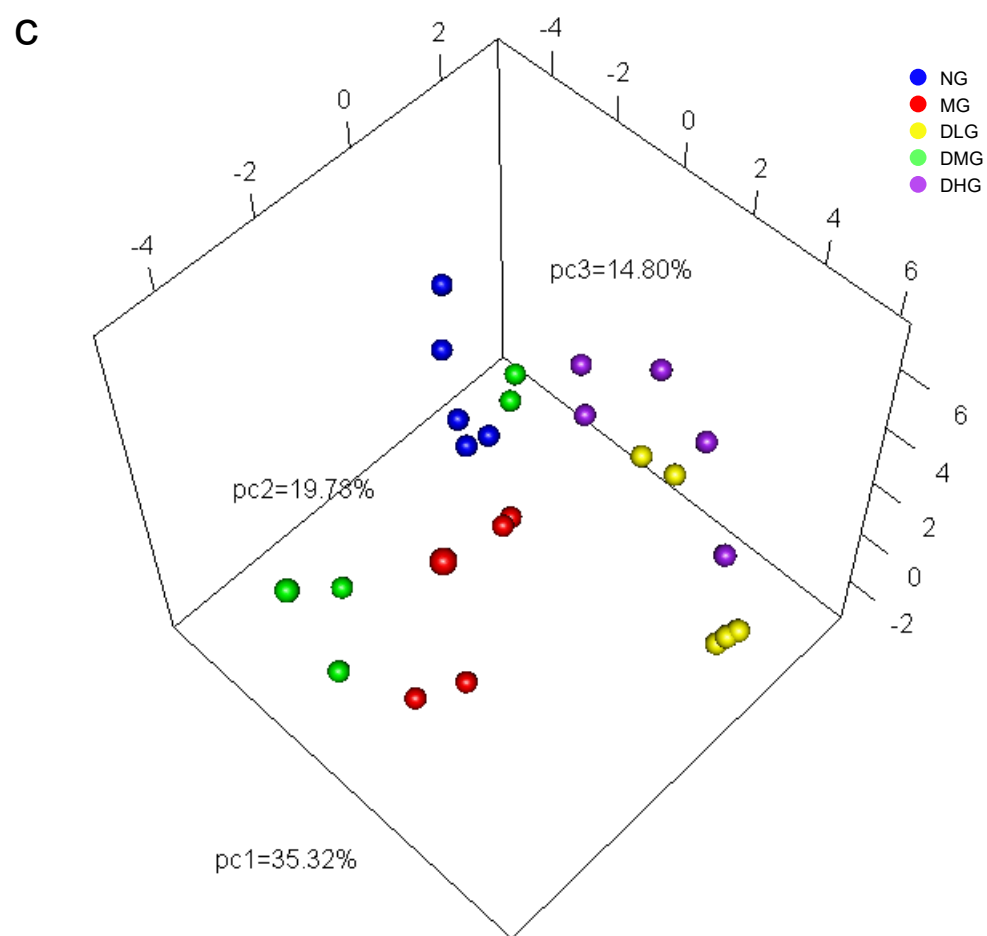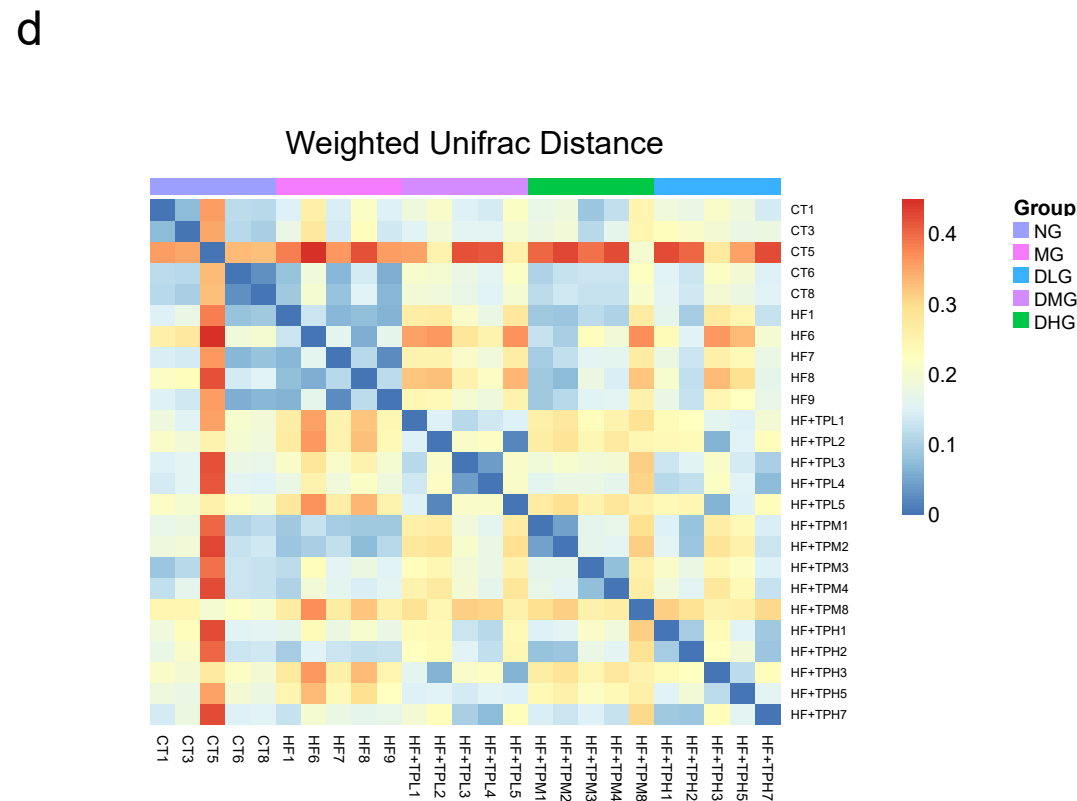

Supplement: Supplementary file 1 [file jmb-34-12-2637-supple.pdf]
